# Machine learning‐based model constructed from ultrasound radiomics and clinical features for predicting HER2 status in breast cancer patients with indeterminate (2+) immunohistochemical results

**DOI:** 10.1002/cam4.6946

**Published:** 2024-01-17

**Authors:** Meiying Yan, Jincao Yao, Xiao Zhang, Dong Xu, Chen Yang

**Affiliations:** ^1^ Department of ultrasound, Zhejiang Cancer Hospital Hangzhou Institute of Medicine (HIM), Chinese Academy of Sciences Hangzhou China; ^2^ Zhejiang Chinese Medical University Hangzhou China; ^3^ Department of ultrasound the First People's Hospital of Hangzhou Lin'an District Hangzhou China

**Keywords:** algorithm, breast cancer, HER2, radiomics, ultrasound

## Abstract

**Background:**

We aimed to predict human epidermal growth factor receptor 2 (HER2) 2+ status in patients with breast cancer by constructing and validating machine learning models utilizing ultrasound (US) radiomics and clinical features.

**Methods:**

We analyzed 203 breast cancer cases immunohistochemically determined as HER2 2+ and used fluorescence in situ hybridization (FISH) as the confirmation method. From each case, the study analyzed 840 extracted radiomics features and 11 clinicopathologic features. Cases were randomly split into training (*n* = 141) and validation sets (*n* = 62) at a 7:3 ratio. Univariate logistic regression analysis was first performed on the 11 clinicopathologic characteristics. The least absolute shrinkage and selection operator (LASSO) and decision tree (DT) techniques were employed for post‐feature selection. Finally, 19 radiomics features were utilized in logistic regression (LR) and Naive Bayesian (NB) classifiers. Model performance was gauged using the area under the receiver operating characteristic curve (AUC), accuracy, sensitivity, and specificity.

**Results:**

Our models exhibited notable diagnostic efficacy in differentiating HER2‐positive from negative breast cancer cases. In the validation sets, the LR model outperformed the NB model with an AUC of 0.860 and accuracy of 83.8% compared to NB's AUC of 0.684 and accuracy of 79.0%. The LR model demonstrated higher sensitivity (92.3% vs. 46.2%) while the NB model had a better specificity (91.8% vs. 63.3%) in the validation set.

**Conclusions:**

Machine learning models grounded on radiomics efficiently predicted IHC HER2 2+ status in breast cancer patients, suggesting potential enhancements in clinical decision‐making for treatment and management.

## 1 INTRODUCTION

Breast cancer is a heterogeneous tumor that can be classified into four major molecular subtypes: luminal‐like (luminal A and luminal B), HER2‐positive, and basal‐like.^1^ The prognosis varies significantly among these molecular subtypes, prompting extensive research into the molecular diagnosis of breast cancer.^2^ HER2 overexpression, which arises from the amplification of the ERBB2/neu proto‐oncogene located on the centromere of chromosome 17 (CEN17), is found in approximately 15%–20% of breast cancers.^3^ HER2‐positive breast cancers are notably aggressive, prone to early recurrence, and linked to a poor prognosis.^4^, ^5^ Despite this, treatments such as trastuzumab (Herceptin) effectively target HER2 protein, benefiting affected patients.^6^ HER2 status is primarily assessed by immunohistochemistry (IHC). According to the ASCO/CAP guidelines, IHC results are categorized as negative (0 or 1+), equivocal (2+), or positive (3+). For the equivocal IHC HER2 (2+) results, a follow‐up with in situ hybridization (ISH), mainly fluorescence in situ hybridization (FISH), is required.^7^ HER2 expression, however, has shown heterogeneity within tumors in almost 40% of cases.^8^, ^9^ Manual HER2 status determination is cumbersome and prone to error, with diagnostic variability among pathologists leading to misdiagnosis in a significant number of cases.^10^, ^11^ Preoperative core needle biopsy (CNB) samples only a fraction of the tumor, with concordance rates between CNB and postoperative histopathology ranging between 81% and 97%.^12^, ^13^, ^14^ Furthermore, changes in HER2 expression are observed in 20%–40% of patients undergoing neoadjuvant chemotherapy.^15^, ^16^ The heterogeneity of HER2 within tumors has been identified as an independent factor linked with inadequate response to neoadjuvant chemotherapy in HER2‐positive patients.^17^ Consequently, enhancing the accuracy and reproducibility of HER2 assessment is crucial. In current clinical practice, real‐time assessment of tumor HER2 expression through multiple preoperative CNBs presents challenges due to the intra‐tumor heterogeneity of HER2. This makes achieving consistent clinical detection and tailoring individualized treatments difficult.^18^ While ISH testing offers a potential solution, its high cost, time‐intensive nature, potential delay in treatment, and limited common use make it less viable. Essentially, there's a crucial need for an accurate, convenient, and non‐invasive method to predict HER2 expression status, facilitating tailored breast cancer treatment to enhance patient prognosis.

Clinical diagnosis of breast tumors relies on imaging examinations, including mammography, magnetic resonance imaging (MRI), and ultrasonography (US). While mammography is essential for breast cancer screening in developed countries, its efficacy diminishes for dense breast tissue.^19^, ^20^ MRI boasts high sensitivity but may be inaccessible due to its cost. In contrast, US offers real‐time, multi‐angle evaluations, is non‐invasive, and radiation‐free, making it ideal for diagnosing breast diseases, especially in dense glands.^21^, ^22^ A form of artificial intelligence (AI) called machine learning (ML), which has recently gained popularity in medicine, uses features from sample data to identify patterns. This has paved the way for computer‐aided diagnosis (CAD) systems in breast cancer.^23^ Currently, numerous ML models are employed to construct CAD systems for breast cancer, such as Naive Bayesian (NB), support vector machine (SVM), and logistic regression (LR). These techniques facilitate data categorization and prediction based on specific features extracted from medical images.^24^ Moreover, ML is also extensively utilized in breast cancer bioinformatics analysis.^25^, ^26^, ^27^ Notably, imaging has been identified as a means to capture tumor biology at genetic and cellular levels.^28^ Some studies have discovered correlations between imaging features and HER2‐positive breast cancer subtypes. For instance, one study found a correlation between extracted peritumor MRI imaging radiomics features and HER2 expression in breast cancer.^29^ Numerous more investigations revealed a link between radiomics features taken from breast mammography and MRI images and HER2‐positive subtypes of breast cancer.^30^, ^31^

In light of the aforementioned findings, our study aimed to investigate the relationship between clinicopathologic factors, US radiomics, and HER2 status. Furthermore, we sought to construct an optimal machine learning model to assist in diagnosing patients with indeterminate IHC results.

## 2 METHODS

### 2.1 Patients and cohorts

Between January 1, 2017, and December 31, 2017, clinical and image data were retrospectively retrieved from the Picture Archiving and Communication System (PACS) at Zhejiang Cancer Hospital. At the hospital, all of the enrolled patients underwent breast surgery. This retrospective study did not require informed consent, guaranteeing that the data were analyzed anonymously. The final results did not include any personal identification. The study was approved by the Zhejiang Cancer Hospital's Institutional Ethics Committee (IRB‐2022‐185).

### 2.2 Pathology assessment

IHC staining scores defined by the College of American Pathologists and the American Society of Clinical Oncology (CAP/ASCO) were used to assess HER2 protein levels in breast cancer tissue samples,^32^ scores 0/1+ denoted “HER2‐negative,” 2+ as “HER2 indeterminate,” and 3+ as “HER2‐positive.” FISH determined the final HER2 expression, with a HER2/CEN‐17 ratio <2 indicating non‐amplification and ≥2 indicating amplification.

Lesions were enrolled using the following criteria. Inclusion criteria encompassed: (1) breast cancer confirmation by postoperative pathology; (2) postoperative specimen IHC results indicating HER2 2+; (3) definitive verification of HER2 status by FISH with clear results; (4) breast ultrasonography conducted within 2 weeks before surgery; (5) clear US images with identifiable lesions. Exclusion criteria were: (1) incomplete clinical data or subpar US quality; (2) lack of postoperative IHC or FISH HER2 status verification, cases with heterogeneous FISH results, or IHC score of 2+ without subsequent FISH testing; (3) preoperative neoadjuvant chemotherapy or radiotherapy; (4) multifocal lesions. Out of 203 female patients that fit the criteria, 161 were HER2‐negative, and 42 were HER2‐positive, with a median age of 53 (range 26–85) years. The dataset was split into a 7:3 ratio, designating 141 patients for training (112 HER2‐negative; 29 HER2‐positive) and 62 for validation (49 HER2‐negative; 13 HER2‐positive). We collected clinical data that included age, menopausal status, T stage (T1 for tumor size ≤20 mm; T2 for 20 mm < tumor size ≤50 mm; T3 for tumor size >50 mm), laterality and quadrant of the breast lesion, family tumor history, levels of estrogen receptor (ER), progesterone receptor (PR), Ki‐67 expression, molecular subtype, and axillary lymph node (ALN) status. All of the patients had either invasive ductal carcinoma (IDC) or ductal carcinoma in situ (DCIS).

### 2.3 US images acquisition and segmentation

**FIGURE 1:**
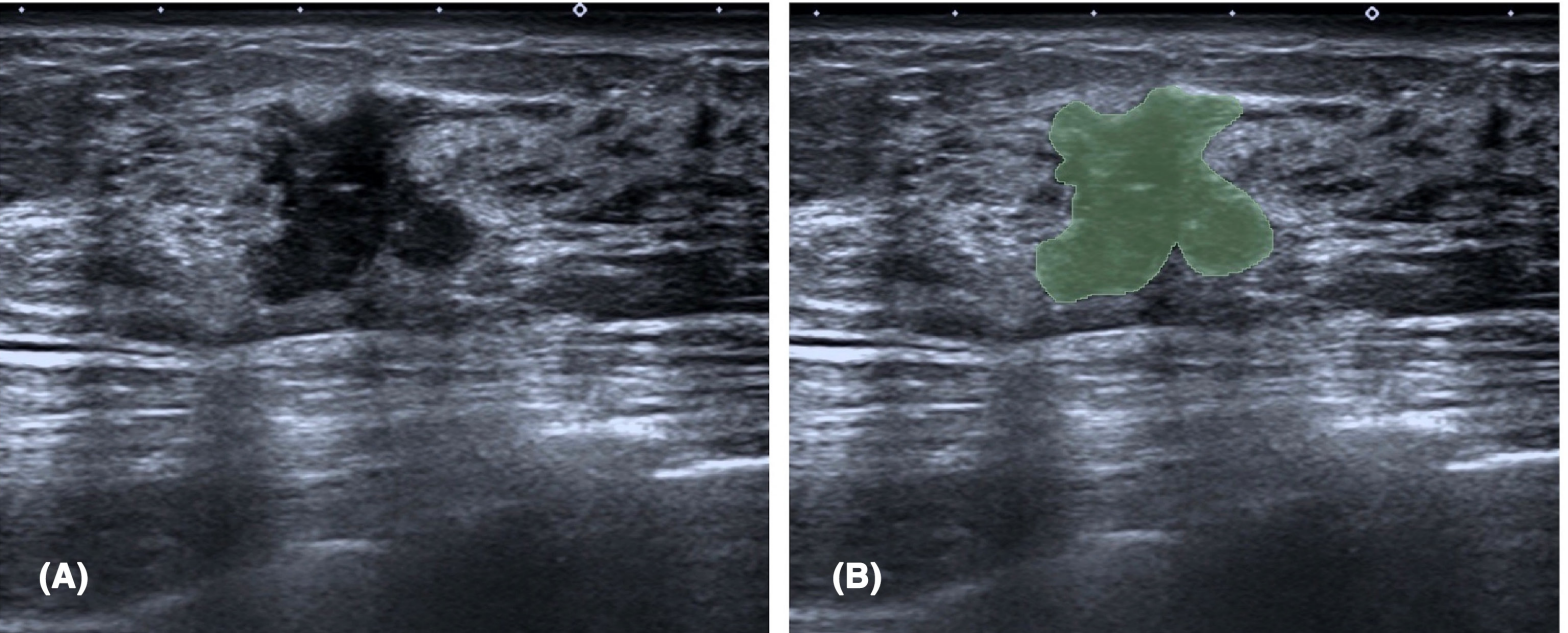
(A) 2D grayscale ultrasound image of breast lesion. (B) Manually outline the region of interest using software 3D slicer.

US evaluations utilized various instruments: LOGIQ E9, Toshiba Aplio 400, Philips iU22, and Siemens S 2000. For the conventional US examinations, a high‐frequency probe model was adopted. Preoperative 2D US images were compiled, and the 3D slicer (version 5.0.3) platform^33^ was employed for analysis. The image parameters were consistent across patients, with a gain set at approximately 50%, an image depth ranging from 3 to 7 cm, and focusing parallel to the lesion. The imaging process ensured clear visualization of the largest section of the lesion, and all ultrasound images were stored in digital imaging and communications in medicine (DICOM) format. The region of interest (ROI) for each tumor was identified by manually outlining the tumor on the transverse view of the 2D grayscale US image in DICOM format, as illustrated in Figure 1. A junior radiologist initially marked the contours, followed by verification by a senior radiologist. Discrepancies were resolved through consensus, with both parties blinded to patient details.

### 2.4 Extraction of radiomics features

Using the SlicerRadiomics plug‐in,^34^ 840 radiomics features across seven categories were initially extracted. These encompassed a total of the following: (1) first‐order statistics, (2) gray‐level co‐occurrence matrix (GLCM) parameters, (3) gray‐level difference method (GLDM) parameters, (4) gray‐level run‐length matrix (GLRLM) parameters, (5) gray‐level size zone matrix (GLSZM) parameters, (6) neighborhood gray‐tone difference matrix (NGTDM) parameters, and (7) shape‐based (2D) parameters.

### 2.5 Features selection and models construction

**FIGURE 2:**
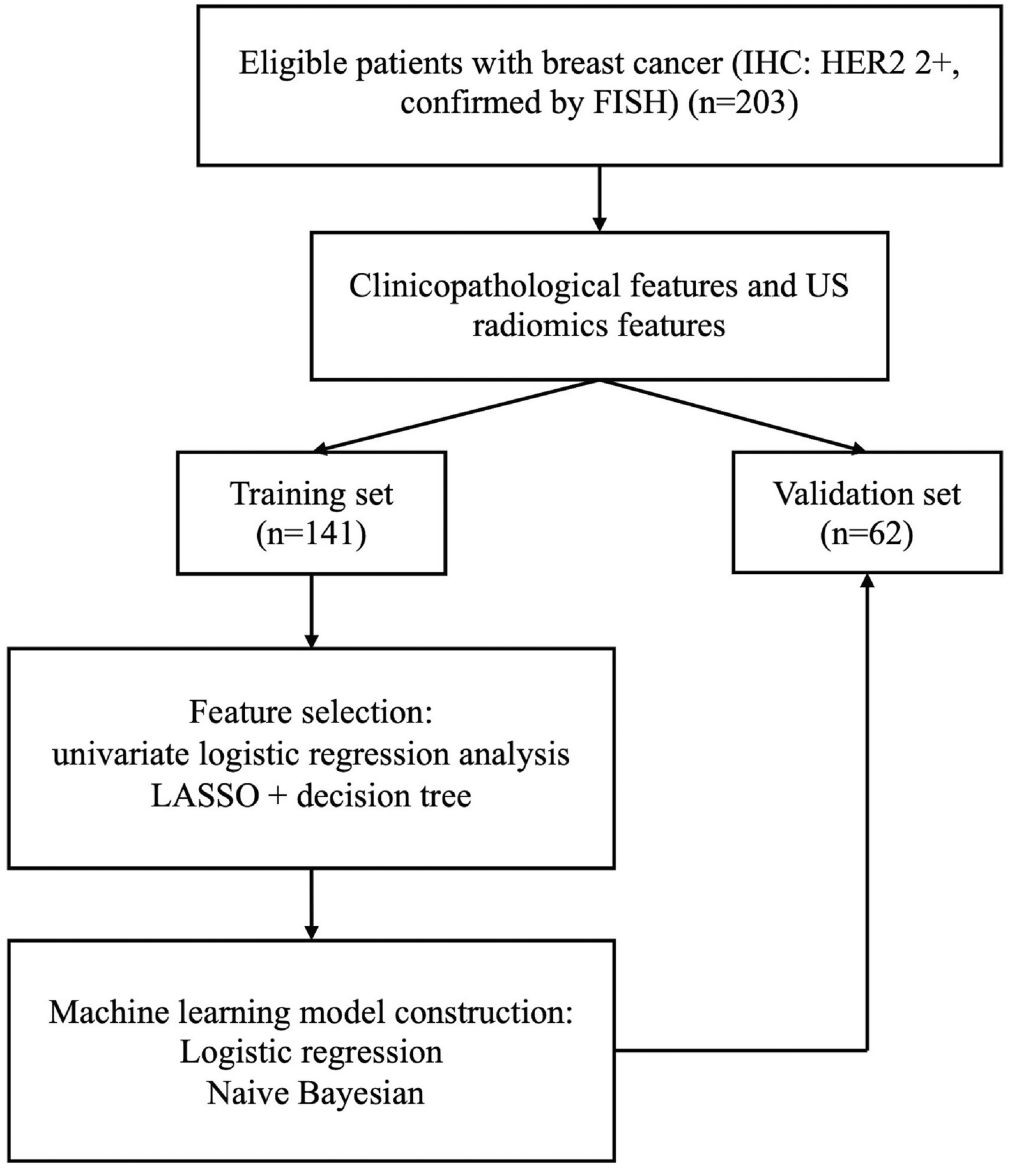
Flowchart of the overall process of constructing the machine learning models for predicting HER2 2+ status.

**FIGURE 3:**
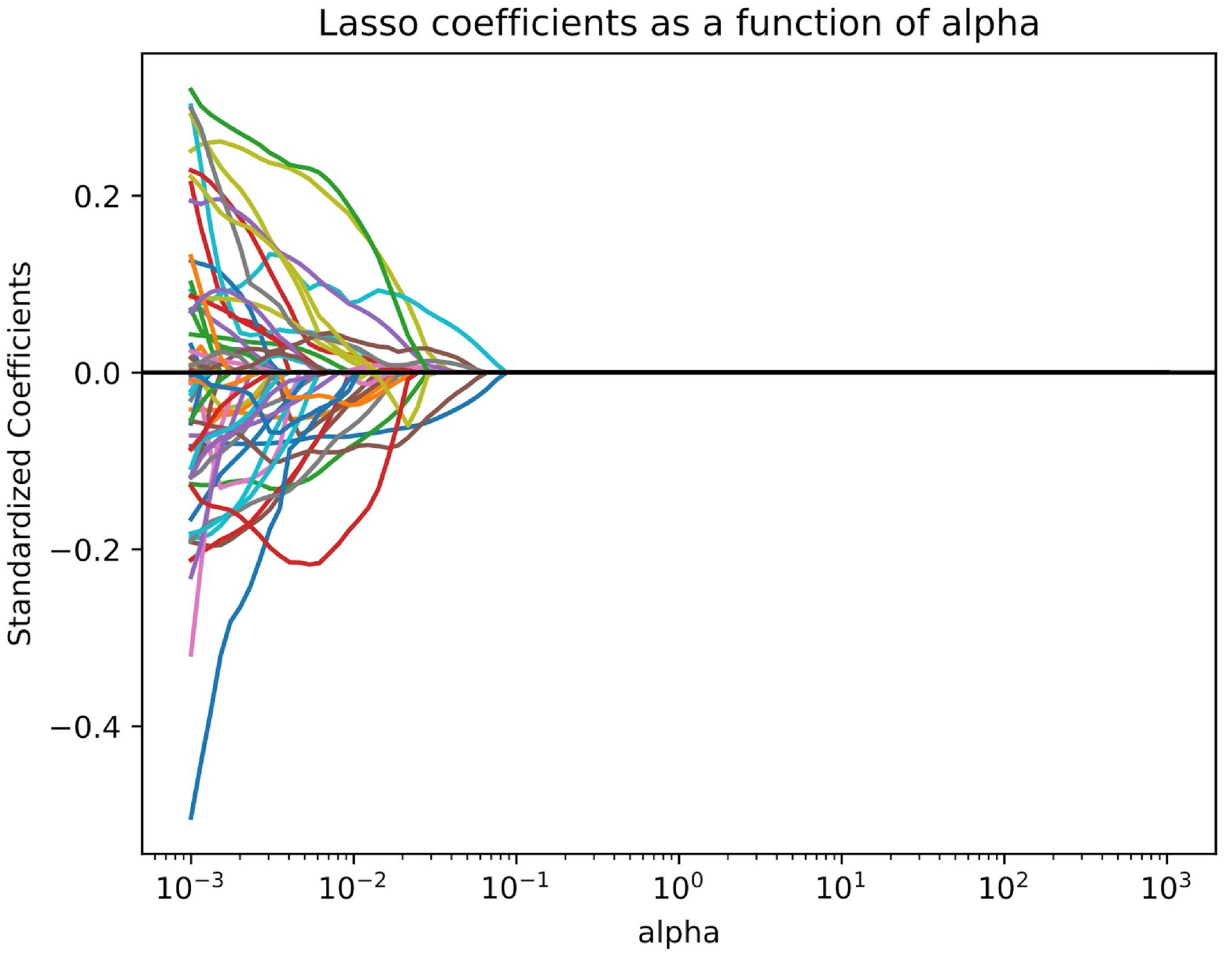
LASSO coefficient solution path of the 86 features. 86 features with non‐zero coefficients were selected from 841 features.

**FIGURE 4:**
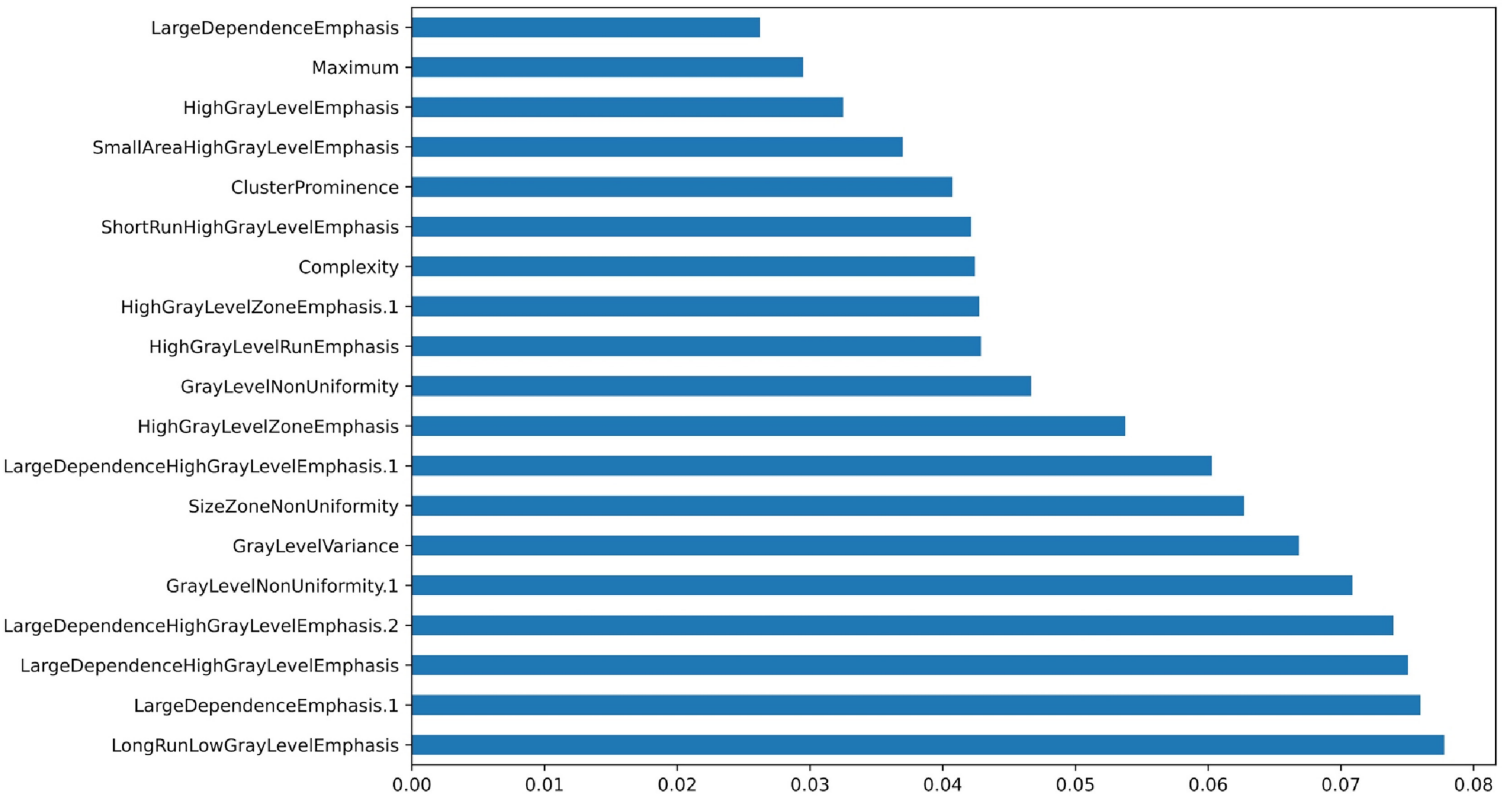
Important parameters for the prediction of HER2 2+ status (19 features).

The model's workflow is depicted in Figure 2. We first screened the training set using univariate logistic regression analysis on 11 clinicopathologic features: age (both as a continuous variable and with a cut‐off at 50 years), menopausal status, family history, T stage, laterality and quadrant of the breast lesion, expression of ER, PR, and Ki‐67, and ALN metastasis status. The clinicopathologic features identified during screening were subsequently subjected to data downscaling and feature screening, in conjunction with ultrasonographic radiomics features. Z‐score and mean normalization methods were employed to standardize the respective characteristics of each patient. Feature selection employed two strategies for reproducibility. The least absolute shrinkage and selection operator (LASSO) was employed to determine the strongest predictive features for HER‐2 status from the set of training data (Figure 3), and the Decision Tree (DT) calculated feature importance. The LASSO is a regularization algorithm. In contrast to ridge regression, the LASSO algorithm incorporates the L1 criterion as a penalty function into the cost function of standard linear regression. It iteratively updates the values of the weight coefficients until an optimal solution is reached. This process involves compressing the weight coefficients of unimportant features to zero, facilitating feature selection.^35^ The appropriate LASSO α parameters were chosen utilizing 10‐fold cross‐validation on the training set. From 841 features, 86 with non‐zero coefficients were screened. After employing the LASSO method, DT is utilized to further screen features. The DT construction process forms a high‐resolution feature subset by ranking features with information gain, adding top‐ranked features as nodes, halting tree growth to prevent overfitting, implementing pruning for size reduction and overfitting mitigation, using a default 25% confidence threshold, determining leaf node characteristics by majority rule, handling misclassifications in small leaf node samples, and generating if‐then rules from the final decision tree. This ensures capturing relevant features while avoiding overfitting.^36^, ^37^ Ultimately, we selected 19 US radiomics features for ML model construction, as depicted in Figure 4. The most robust and significant selected features were put into logistic regression (LR) and Naive Bayesian (NB) classifiers, validation for the prediction of HER2 status. The performance of the ML models was evaluated using the receiver operating characteristic (ROC) curve, with attention to the area under the curve (AUC) for both training and validation sets. Additionally, accuracy (ACC), sensitivity (SEN), and specificity (SPE), positive predictive value (PPV), and negative predictive value (NPV) metrics were computed for both datasets. AUC values between different classifiers were compared, and *p* values were calculated using DeLong's test.

### 2.6 Statistical analysis

SPSS Statistics (version 26.0) and Python (version 3.11.3) were implemented to analyze the data. Numeric data is presented as mean ± SD. The independent sample t‐test or chi‐square test was used to evaluate continuous and categorical variables. A *p* value below 0.05 was deemed statistically significant.

## 3 RESULTS

### 3.1 Patient characteristics

Two hundred three patients with breast cancer were examined for HER2 2+ status by IHC. Subsequent clarification using FISH categorized the patients into HER2‐negative (*n* = 161) and HER2‐positive groups (*n* = 42). The comparative clinicopathological features of the two groups are detailed in Table 1. Notably, the HER2‐positive group exhibited a significantly higher Ki67 expression index compared to the HER2‐negative group (*p* = 0.027). Moreover, molecular subtypes varied significantly between the two groups (*p* < 0.001). The fundamental clinicopathological characteristics of the patients, split between the training and validation sets, are demonstrated in Table 2. No notable differences were observed between the two sets concerning age, menopausal status, family tumor history, HER2, ER, PR, Ki‐67 expression levels, T stage, lateralization and quadrant of breast lesions, and molecular subtypes (all *p* > 0.05). However, the training set had a higher incidence of axillary invasion compared to the validation set, with this difference being statistically significant (*p* = 0.031).

**TABLE 1:** Clinicopathological characteristics of IHC HER2 2+ patients with breast cancer (*n* = 203).

Characteristics	FISH status		*p* Value
	HER2‐negative	HER2‐positive	
	(*n* = 161)	(*n* = 42)	
Age (years, mean ± SD)	52.6 ± 9.8	51.02 ± 11.7	0.361
Age (years) (%)			
<50	65 (40.4)	16 (38.1)	0.788
≥50	96 (59.6)	26 (61.9)	
Menopause (%)			
Yes	88 (54.7)	22 (52.4)	0.792
No	73 (45.3)	20 (47.6)	
Family history (%)			
Yes	33 (20.5)	12 (28.6)	0.262
No	128 (79.5)	30 (71.4)	
T stage (%)			
T1	66 (41.0)	23 (54.8)	0.201
T2	90 (55.9)	17 (40.5)	
T3	5 (3.1)	2 (4.8)	
Laterality of lesion (%)			
Left	79 (49.1)	24 (57.1)	0.351
Right	82 (50.9)	18 (42.9)	
Quadrant of lesion (%)			
Upper outer	75 (46.6)	22 (54.2)	0.190
Lower outer	17 (10.6)	9 (21.4)	
Lower inner	13 (8.1)	3 (7.1)	
Upper inner	37 (23.0)	6 (14.3)	
Central	19 (11.8)	2 (4.8)	
ER (%)			
Positive	129 (80.1)	31 (73.8)	0.372
Negative	32 (19.9)	11 (26.2)	
PR (%)			
Positive	121 (75.2)	26 (61.9)	0.087
Negative	40 (24.8)	16 (38.1)	
Ki‐67 (%)			
≥14	115 (71.4)	37 (88.1)	**0.027**
<14	46 (28.6)	5 (11.9)	
Molecular subtype (%)			
Luminal‐like	129 (80.1)	32 (76.2)	**<0.001**
Triple negative	32 (19.9)	0 (0.0)	
HER2‐amplified	0 (0.0)	10 (23.8)	
ALN metastasis status (%)			
Positive	81 (50.3)	24 (57.1)	0.430
Negative	80 (49.7)	18 (42.9)	

*Note*: The bold values mean *p* value <0.05, representing significant differences.

Abbreviations: ALN, axillary lymph node; ER, estrogen receptor; FISH, fluorescence in situ hybridization; HER2, human epidermal growth factor receptor‐2; PR, progesterone receptor; SD, standard deviation.

**TABLE 2:** Clinicopathological characteristics of IHC HER2 2+ patients with breast cancer in the training and validation sets (*n* = 203).

Characteristics	Training set (*n* = 141)	Validation set (*n* = 62)	*p* Value
Age (years, mean ± SD)	52.0 ± 10.4	53.1 ± 9.8	0.456
Age (years) (%)			
<50	62 (44.0)	19 (30.6)	0.074
≥50	79 (56.0)	43 (69.4)	
Menopause (%)			
Yes	73 (51.8)	37 (59.7)	0.298
No	68 (48.2)	25 (40.3)	
Family history (%)			
Yes	27 (19.1)	18 (29.0)	0.118
No	114 (80.9)	44 (71.0)	
T stage (%)			
T1	59 (41.8)	30 (48.4)	0.468
T2	78 (55.3)	29 (46.8)	
T3	4 (2.8)	3 (4.8)	
Laterality of lesion (%)			
Left	76 (53.9)	27 (43.5)	0.174
Right	65 (46.1)	35 (56.5)	
Quadrant of lesion (%)			
Upper outer	65 (46.1)	32 (51.6)	0.476
Lower outer	22 (15.6)	4 (6.5)	
Lower inner	11 (7.8)	5 (8.1)	
Upper inner	28 (19.9)	15 (24.2)	
Central	15 (10.6)	6 (9.7)	
ER (%)			
Positive	113 (80.1)	47 (75.8)	0.486
Negative	28 (19.9)	15 (24.2)	
PR (%)			
Positive	103 (73.0)	44 (71.0)	0.760
Negative	38 (27.0)	18 (29.0)	
Ki‐67 (%)			
≥14	107 (75.9)	45 (72.6)	0.617
<14	34 (24.1)	17 (27.4)	
HER2 (%)			
Positive	29 (20.6)	13 (21.0)	0.948
Negative	112 (79.4)	49 (79.0)	
Molecular subtype (%)			
Luminal‐like	113 (80.1)	48 (77.4)	0.168
Triple negative	19 (13.5)	13 (21.0)	
HER2‐amplified	9 (6.4)	1 (1.6)	
ALN metastasis status (%)			
Positive	80 (56.7)	25 (40.3)	**0.031**
Negative	61 (43.3)	37 (59.7)	

*Note*: The bold values mean *p* value <0.05, representing significant differences.

Abbreviations: ALN, axillary lymph node; ER, estrogen receptor; FISH, fluorescence in situ hybridization; HER2, human epidermal growth factor receptor‐2; PR, progesterone receptor; SD, standard deviation.

### 3.2 Feature dimensionality reduction and screening

For each patient, 840 quantitative US radiomics features were retrieved. Out of the 840 radiomics features, calculations were performed for 162 first‐order features, 216 GLCM parameters, 126 GLDM parameters, 144 GLRLM parameters, 144 GLSZM parameters, 45 NGTDM parameters, and 3 shape‐based parameters. Univariate logistic regression indicated a significant difference in PR between the HER2‐positive and negative groups within the training set (*p* = 0.017) as shown in Table 3. Following this, we combined 840 radiomics features with the clinical factor PR, resulting in 841 features, for further analysis. LASSO screening identified 86 features with non‐zero coefficients. Following this, we applied DT analysis to determine the relative importance of each feature, primarily leveraging the information gain rate. This analysis culminated in the selection of 19 crucial radiomics features. Figure 4 depicts these 19 key features vital for HER2 status prediction. Notably, ML approaches enabled the successful development of HER2 prediction models using the training set, and their efficacy was subsequently validated by the validation set.

**TABLE 3:** Results of univariate logistic regression analysis of clinicopathologic factors for predicting HER2 positivity in the training set (*n* = 141).

Variables	OR	95% CI	*p* Value
Age (years, mean ± SD)	0.979	0.940–1.019	0.300
Age (years)	0.957	0.421–2.177	0.917
Menopause	0.838	0.370–1.899	0.673
Family history	1.464	0.550–3.893	0.445
T stage	0.574	0.265–1.244	0.160
Laterality of lesion	0.786	0.344–1.796	0.568
Quadrant of lesion	0.969	0.708–1.326	0.844
ER	0.454	0.179–1.149	0.096
PR	0.354	0.150–0.833	**0.017**
Ki‐67	2.287	0.735–7.116	0.153
ALN metastasis status	0.651	0.287–1.477	0.304

*Note*: The bold values mean *p* value <0.05, representing significant differences.

Abbreviations: ALN, axillary lymph node; CI, confidence interval; ER, estrogen receptor; HER2, human epidermal growth factor receptor‐2; OR, odds ratio; PR, progesterone receptor.

### 3.3 Machine learning model performance for prediction of HER2 status

**FIGURE 5:**
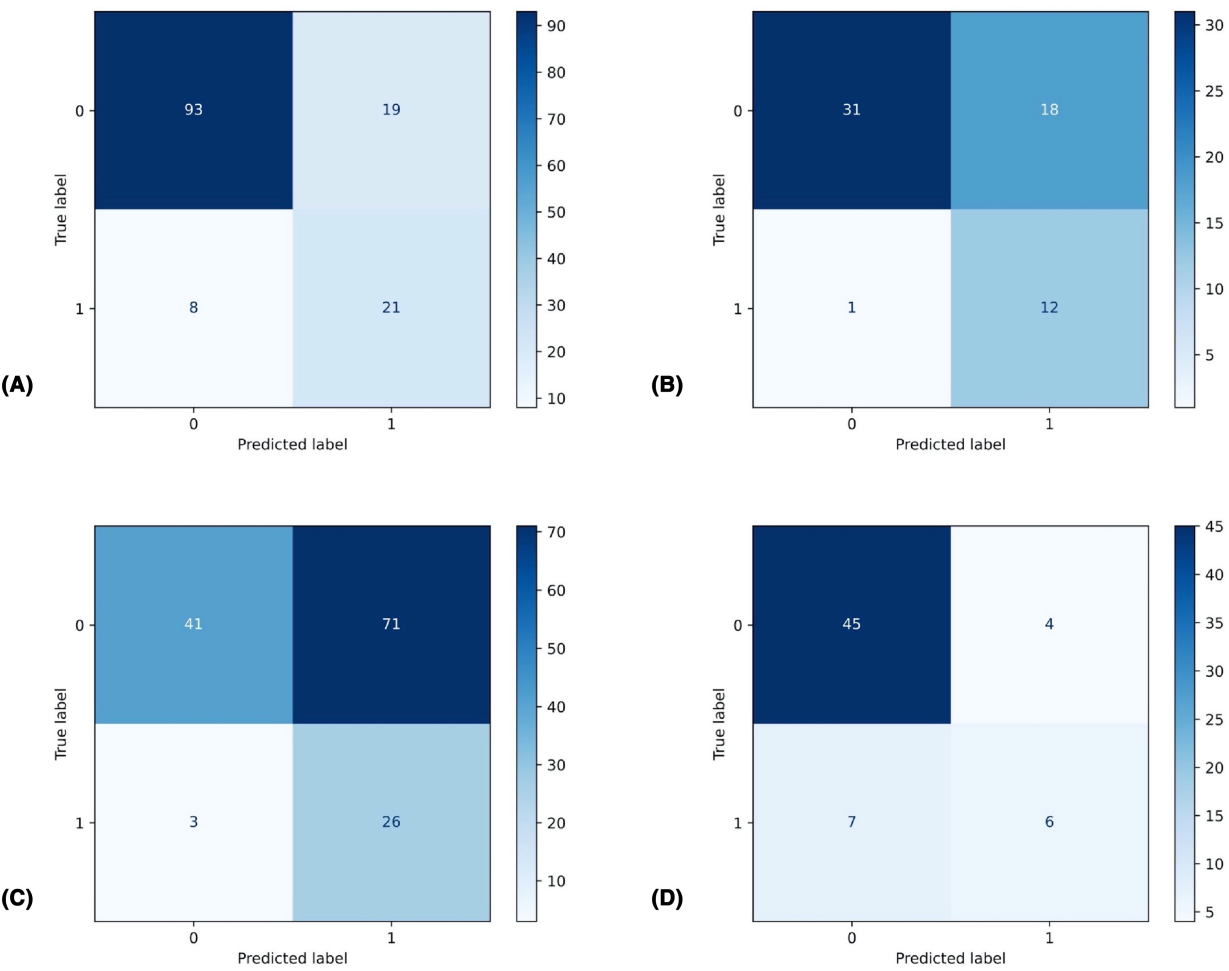
Results of Confusion matrix. (A) Prediction results of the logistic regression (LR) model on the training set; (B) prediction results of the LR model on the validation set; (C) prediction results of the Naive Bayesian (NB) model on the training set; (D) prediction results of the NB model on the validation set.

**FIGURE 6:**
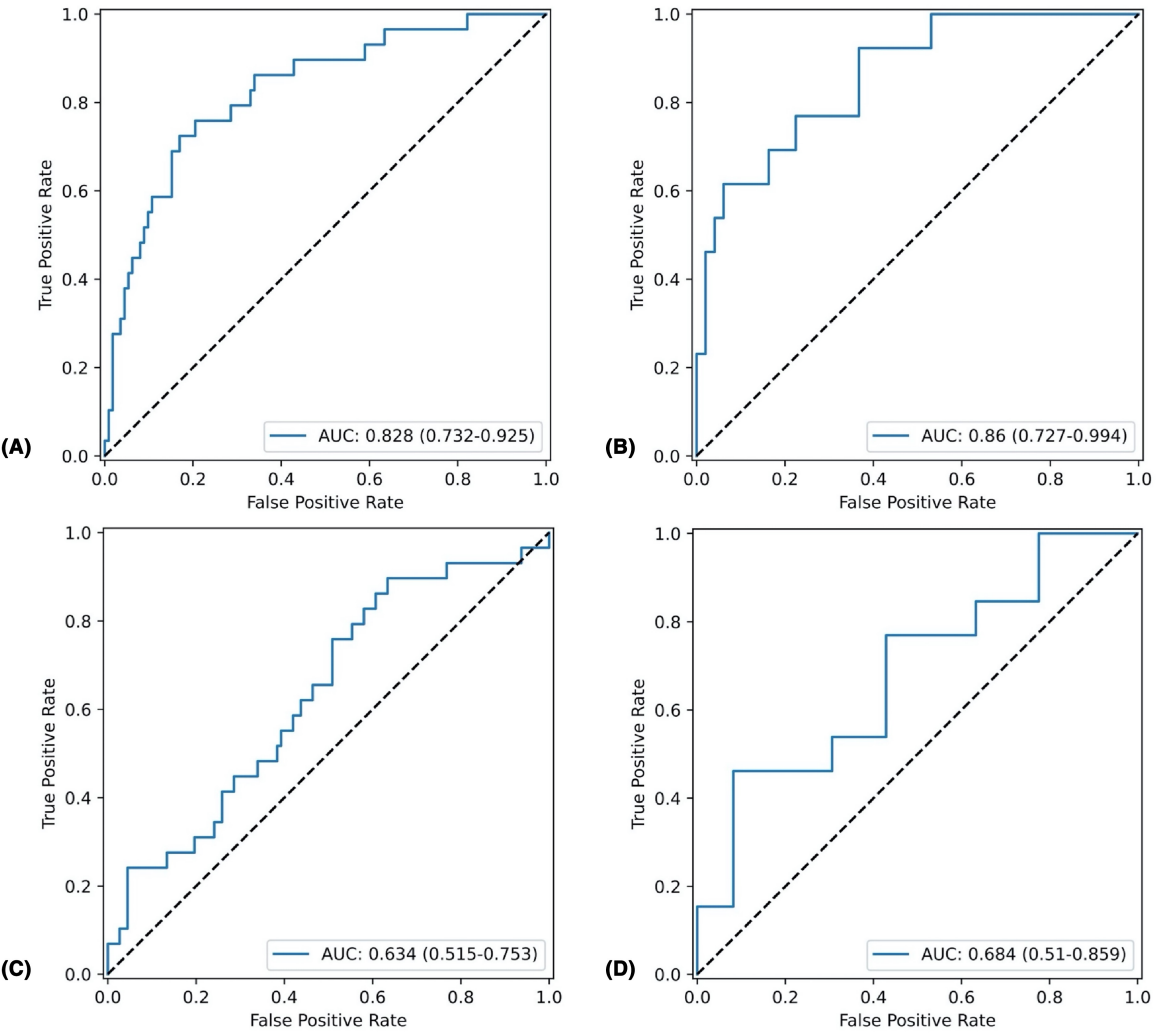
ROC curves of the machine learning model for the prediction of HER2 2+ status. (A) Training set of the LR model; (B) validation set of the LR model; (C) training set of the NB model; (D) validation set of the NB model.

**FIGURE 7:**
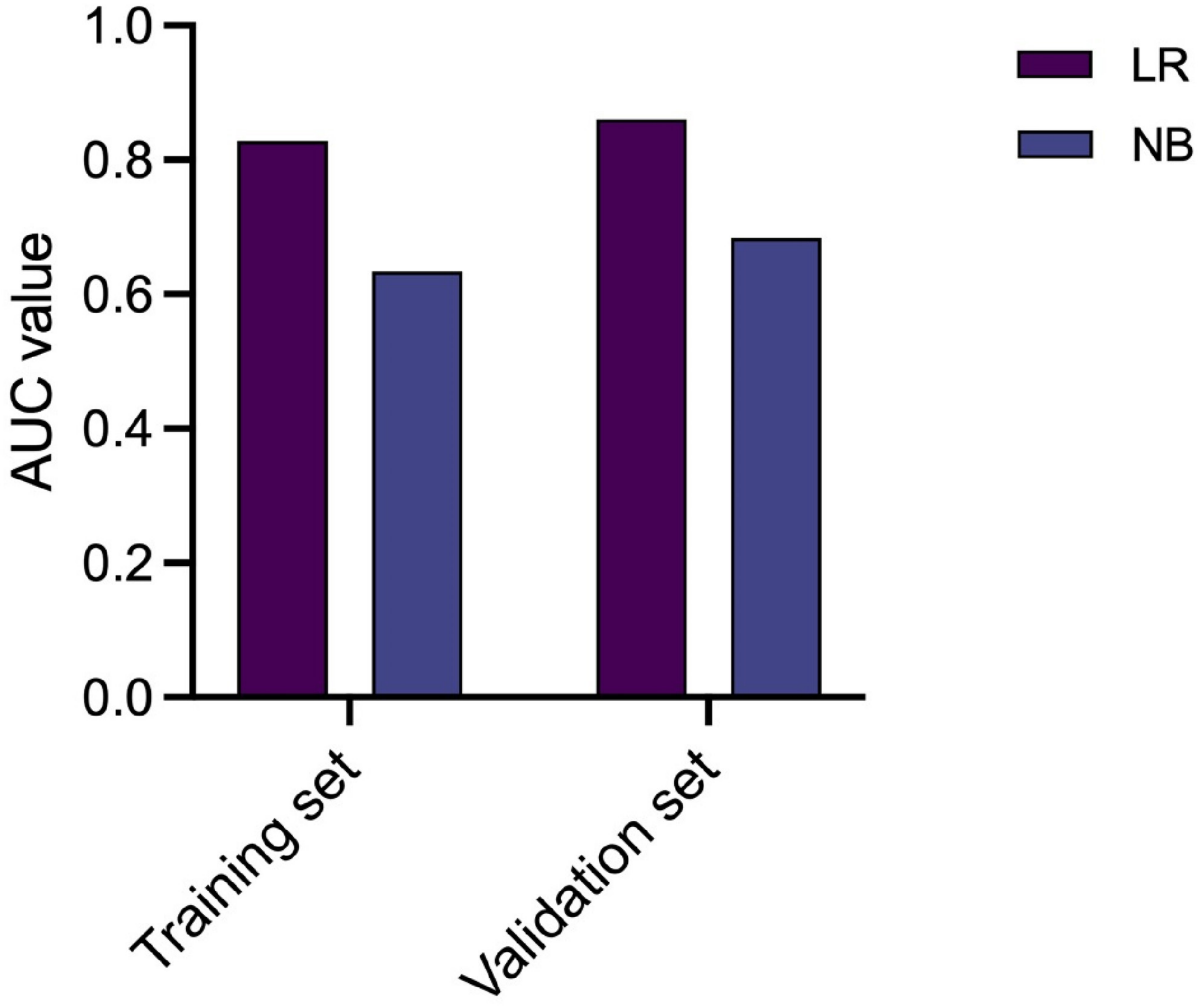
Area under the curve (AUC) values for LR and NB models for training and validation sets.

Table 4 presents the predictive performance of both the LR and NB models. Figure 5 illustrates the results of the confusion matrix, while Figure 6 displays the ROC analysis outcomes. Figure 7 provides the AUC values of the models for both training and validation sets. In comparing the performance metrics of the LR model and the NB model, it was observed that the LR model generally outperformed the NB model across both training and validation data sets. Specifically, within the validation set, the LR model demonstrated a commendable performance, achieving an AUC value of 0.860, an accuracy rate of 83.8%, a sensitivity of 92.3%, a PPV of 40.0%, and a NPV of 96.9%. The AUC value of the LR model was significantly higher than that NB model (DeLong test, *p* = 0.031) in the validation set. These results underscore the LR model's robustness and its efficacy in accurately classifying and predicting outcomes based on the given data. In contrast, while the NB model exhibited a lower performance in terms of AUC, accuracy, and sensitivity, it was noteworthy for its impressive specificity, which stood at 91.8% in the validation set. This indicates that the NB model possesses a high capability to correctly identify negative cases, making it a valuable tool in contexts where the cost of false positives is high, and specificity is of paramount importance.

**TABLE 4:** The results of model performance evaluation (*n* = 203).

Model	Training set (*n* = 141)						Validation set (*n* = 62)					
	AUC (95% CI)	ACC (%)	SEN (%)	SPE (%)	PPV (%)	NPV (%)	AUC (95% CI)	ACC (%)	SEN (%)	SPE (%)	PPV (%)	NPV (%)
LR	0.828 (0.732–0.925)	82.9	72.4	83.0	52.5	92.1	0.860 (0.727–0.994)	83.8	92.3	63.3	40.0	96.9
NB	0.634 (0.515–0.753)	79.4	89.7	36.6	30.4	85.9	0.684 (0.510–0.859)	79.0	46.2	91.8	29.0	87.1

Abbreviations: ACC, accuracy; AUC, area under the curve; CI, confidence interval; LR, logistic regression; NB, Naive Bayesian; NPV, negative predictive value; PPV, positive predictive value; ROC, receiver operating characteristic; SEN, sensitivity; SPE, specificity.

## 4 DISCUSSION

Patients with HER2‐positive breast cancer commonly respond favorably to neoadjuvant chemotherapy with the HER2‐targeted agent trastuzumab, leading to improved disease‐free survival and overall survival.^38^, ^39^ Thus, HER2 status remains pivotal for determining breast cancer prognosis and tailoring optimal treatment strategies. Radiomics involves extracting and analyzing quantitative imaging features from modalities like US and CT. These features, often subtle to human eyes, can be quantified to correlate with the lesion's intrinsic qualities. This correlation enables the imaging phenotype to align with genetic information, aiding in disease diagnosis, individualized treatment strategies, and prognosis prediction.^28^, ^40^, ^41^

FISH was employed in this work to ascertain HER2 2+ status and to assess the efficacy of the variables in classifying HER2 2+ status. We examined the concordance between our ML predictions based on radiomics features and the FISH assay results. A study involving 1951 cases revealed that HER2‐positive patients with breast cancer tended to have a higher Ki‐67 proliferation index,^42^ which aligns with our findings. In our cohort, 88.1% of HER2‐positive patients had a high Ki‐67 proliferation index (37/42; *p* = 0.027). A meta‐analysis of 12 papers discerned a relationship between breast US features and HER2 expression, identifying three US features with significant associations to HER2 overexpression: microcalcifications on US imaging, indications of malignancy on US, and irregular breast mass shapes on US.^43^ Although some correlation emerged, the results were variable and somewhat limited, underscoring the insufficiency of US features alone in comprehensively characterizing lesions, leading us to further investigate radiomics.

Previous studies have documented the diagnostic efficacy of various ML algorithms in the context of breast cancer. LR is advantageous for breast cancer diagnosis with medical imaging radiomics due to its interpretability, efficiency in binary classification, probabilistic outputs, adaptability to linear and non‐linear relationships, low risk of overfitting, efficiency with small datasets, suitability for feature selection, and easy implementation.^44^, ^45^ Jing Zhuo et al.^18^ reported that, in the application of mammography (MG) radiomics, a LR model demonstrated superior performance (AUC = 0.787) compared to the SVM model in predicting the HER2 status of patients with breast cancer. NB is well‐suited for handling high‐dimensional data.^24^ Matthias Benndorf et al.^46^ developed and validated a NB model for breast mass lesions using standardized descriptive terminology (BI‐RADS lexicon) to mitigate variability in practice (AUC = 0.935). Woojae Kim et al.^47^ reported recruiting a total of 679 patients and constructing a nomogram based on Naive Bayes (NB) modeling to predict recurrence within 5 years after breast cancer surgery, achieving an AUC of 0.81.

We deployed two ML models, LR and NB models, based on US radiomics to forecast HER2 status in patients preliminarily diagnosed as HER2 2+ via IHC. Given that IHC can discern HER2‐negative (0/1+) from HER2‐positive (3+) during the initial stages, accurately establishing the eventual status of HER2 2+ is becoming increasingly important in clinical choice‐making. When comparing the predictive performance of both models, the LR model clearly outshined. For the validation set, its accuracy and AUC stood at 83.8% and 0.860 (95% CI: 0.727–0.994) respectively. While the LR model boasted a higher sensitivity of 92.3% in the validation set, the NB model impressively registered a specificity of 91.8%. According to a study conducted by Zejun Jiang et al., AUCs for texture analysis‐based ML models of breast dynamic contrast enhancement‐magnetic resonance (DCE‐MR) images for predicting HER2 2+ patients with breast cancer ranged from 0.808 to 0.865.^48^ However, the sample size of that study was very small, with only 73 cases and 279 texture features and there was no validation set to validate the results. While our models exhibited promise, it's crucial to emphasize their current role as potential adjuncts rather than replacements for FISH testing in differentiating HER2 IHC 2+ breast cancer patients. It may help to classify FISH cases or collaborate with oncologists in formulating initiate treatment plans.

Our research advocates for the potential utility of ML classifiers derived from radiomics features in ultrasound images as a non‐invasive methodology to ascertain HER‐2 status. However, this study is not devoid of limitations. (1) Firstly, this study was a single‐center study and lacked external validation sets. The predictive sample size for this study was relatively small. Multi‐center studies are needed to bolster heterogeneity and model adaptability. (2) Secondly, this study was retrospective, accompanied by sample imbalances and inevitable selection bias, which may impact the results of variable analysis. Future large‐scale and prospective research should be undertaken to mitigate this error. (3) Thirdly, manual ROI delineations may not comprehensively represent lesions, and it is time‐consuming and labor‐intensive. Applying ML models alone cannot achieve the automatic identification and segmentation of lesions. Therefore, the implementation of AI‐automated operating systems will be pursued in the future. (4) We collected only 2D grayscale US images for radiomics analysis; multi‐modal US features, such as US elastography, were not included. This suggests a potential shift toward three‐dimensional imaging modalities, like automated breast ultrasound (ABUS), and apply multi‐modal US features in future endeavors.

The understanding and classification of HER2 are continually evolving. In recent times, the category of “HER2 low” breast cancers has been introduced. These primarily encompass tumors designated as IHC 1+ or 2+ but are ISH‐negative.^49^ Clinically, interpreting HER2 can be challenging when FISH tests indicate HER2 heterogeneity. Accurate classification becomes paramount, especially since tumors with a lower range of HER2 expression might respond favorably to new targeted therapies. In this light, ML models leveraging US images offer promising avenues. They can discern predictive information for IHC HER2 2+ cases, providing valuable insights into the potential HER2 outcome. Furthermore, the ML models we constructed can be employed repeatedly, both pre‐ and post‐treatment, offering guidance for HER2‐targeted therapies. This approach paves the way for tailored treatments and precision medicine in breast cancer care.

In conclusion, our machine learning models, particularly LR, show promise for deciphering indeterminate IHC HER2 (2+) results in breast cancer, offering valuable insights for targeted therapies. While not a replacement for FISH tests, these models enhance decision‐making for individual patient needs. Validation with extensive external datasets is crucial before clinical integration. This research sets the stage for future explorations, including multi‐modal deep learning in upcoming studies.

## AUTHOR CONTRIBUTIONS

**Meiying Yan:** Conceptualization (equal); data curation (equal); formal analysis (equal); methodology (equal); resources (equal); software (equal); validation (equal); writing – original draft (equal); writing – review and editing (equal). **Jincao Yao:** Data curation (equal); formal analysis (equal); methodology (equal); software (equal); validation (equal); visualization (equal). **Xiao Zhang:** Formal analysis (supporting); investigation (supporting); resources (lead); validation (supporting); visualization (supporting). **Dong Xu:** Conceptualization (equal); investigation (equal); project administration (equal); resources (supporting); supervision (equal); validation (equal); writing – review and editing (equal). **Chen Yang:** Conceptualization (equal); funding acquisition (equal); investigation (equal); project administration (equal); supervision (equal).

## FUNDING INFORMATION

Zhejiang Provincial Medical and Health Science and Technology Plan Project (2022KY669 and 2022KY1058); Hangzhou Health Science and Technology Plan Project (B20220443).

## CONFLICT OF INTEREST STATEMENT

There are no conflicts of interest of the authors.

## ETHICS STATEMENT

The study was approved by the Zhejiang Cancer Hospital's Institutional Ethics Committee (IRB‐2022‐185).

## CONSENT

The final results did not include any personal identification.

## Data Availability

The datasets generated during and/or analyzed during the current study are available from the corresponding author on reasonable request.
